# Dosimetry comparison with helical tomotherapy, volumetric modulated arc therapy, and intensity-modulated radiotherapy for grade II gliomas: A single‑institution case series

**DOI:** 10.1515/biol-2022-0550

**Published:** 2023-02-10

**Authors:** Mao Sun, Lu Lu Wang, Shi Qiang Wang, Xin Lin, Wei Zhou

**Affiliations:** Department of Radiotherapy Oncology, Chongqing University Cancer Hospital, Chongqing, 400010, China; Department of Neurooncology Surgery Center, Chongqing University Cancer Hospital, Chongqing, 400010, China

**Keywords:** dosimetry comparison, radiotherapy, gliomas, TOMO, IMRT, VMAT

## Abstract

Radiotherapy is an essential postoperative treatment for grade II gliomas. However, comparative dosimetric studies of different radiotherapy plans for grade II gliomas are still lacking. Therefore, we conducted this case series analysis to compare the dosimetric differences among helical tomotherapy (TOMO), volumetric modulated arc therapy (VMAT), and intensity-modulated radiotherapy (IMRT) for grade II gliomas. To achieve that, seven diagnosed postoperative patients with grade II gliomas were analyzed by computed tomography and then planned with TOMO, VMAT, and IMRT. The plan target volume (PTV) prescribed dose was 50 Gy (daily fraction of 2.0 Gy, 5 days/week). The expected treatment efficiency was measured by monitor units (MUs) scoring. Treatment plans of the patients were compared in the quality of target volumes dosage coverage, the efficiency of dosage delivery, and the dosage exposure of normal adjacent organs at risk (OAR). Differences in each method were measured by utilizing the Nonparametric ANOVA. The study shows that TOMO achieved a significantly higher PTV-D_98%_ (doses received by 98% of the PTV volume) than VMAT and IMRT (50.30 ± 0.13 vs 49.21 ± 0.19, *p* = 0.006; 50.30 ± 0.13 vs 49.78 ± 0.18, *p* = 0.014), while there was no difference in PTV-D_2%_ (doses received by 2% of the PTV volume). IMRT achieved a conformity index (CI) preferably, and TOMO generated a favorable homogeneity index (HI) (*p* < 0.05 for both). The MUs were fewer for VMAT than IMRT and TOMO (294 ± 19, 572 ± 24, 317 ± 97, respectively). IMRT achieved better protection for the lens and brain stems. Our case series study indicated that TOMO, VMAT, and IMRT achieved a comparatively good target dosimetric coverage, and most OARs were protected well. IMRT is not inferior to TOMO and VMAT and is still very suitable for treating most grade II glioma patients.

## 1 Introduction

Gliomas are the most common primary tumor of the intracranial central nervous system. According to the current WHO classification, gliomas are divided into four histological grades [1,2,3]. Low-grade gliomas (LGGs) comprise a heterogeneous group of neoplasms that exhibit substantial variations in biological behavior despite a relatively uniform histology-based classification. Surgery and postoperative adjuvant radiotherapy are considered the mainstream treatment methods for LGGs. Radiation therapy (RT) was reported to be related to the clinical benefits of LGGs [2,4,5]. However, there is still a debate on balancing the efficacy and side effects of RT for LGGs. The potential for late toxicity may offset the tumor local control benefit of RT in patients with glioma. Potential late toxicity is mainly related to the range of radiation and dose of normal brain tissue [6,7]. Therefore, there is an urgent need to find better radio-therapeutic techniques, including treatment plans, target volume (TV), and radiation dose in clinical practice.

Radiotherapy technology has been dramatically improved recently. IMRT is different from previous radiotherapy techniques. It produces non-uniform irradiation by adjusting the intensity of field rays in all directions to achieve high-dose three-dimensional suitable tumor distribution, thus dramatically improving the tumor’s radiation dose. IMRT can decrease the probability of radiation complications by reducing the dose of organs at risk (OAR) and surrounding normal tissues [8]. However, IMRT has a long single irradiation time and a large amount of machine output dose, resulting in a large volume of surrounding normal tissues being exposed to low-dose irradiation. Helical Tomotherapy (TOMO) is the best IMRT system to date, combines IGRT and IMRT into one, using spiral computed tomography (CT) rotation to capture images and treat tumors, and can achieve adaptive radiotherapy (ART) or dose-guided radiotherapy. The TOMO can achieve the IMRT with the best conformance and dose uniformity and uses volume intensity-modulated rotation techniques with non-uniform intensity rays, different (non-uniform) intensity levels, and sufficient field angles (150–250°) to achieve the desired design for measurement in the target area [9]. VMAT is developed based on IGRT technology. It has a new high-precision accelerator, excellent reverse optimization treatment plan software, and precision dose verification equipment to achieve accurate positioning, planning, and treatment. VMAT with gating capability has had increasing adoption in many clinics in China. In this new technique, dose rate, gantry rotation speed, and the leaf motion speed of multi leaf collimators (MLCs) are modulated dynamically during gated beam delivery to achieve highly conformal dose coverage of the target and normal tissue sparing. Finally, the optimal dose distribution of the reverse plan achieves the best quality and improves operational efficiency [4,10]. VMAT has the characteristics of being fast, accurate, and optimum. IMRT, VMAT, and helical tomotherapy are highly conformal radiation techniques widely used in head-and-neck cancers, including gliomas [3,11,12,13]. We must thoroughly understand each technology’s features, advantages, and disadvantages in many new technologies and make an excellent selection to give full play to the value of precision radiotherapy. Recently, radiotherapy techniques comparisons for treating patients with gliomas or high-grade gliomas indicate some potential disadvantages and advantages. However, dosimetric data on various radiotherapy techniques in LGGs are still lacking. This study aims to assess further treatment plans utilizing three radiotherapy techniques, TOMO, VMAT, and IMRT, to treat LGGs.

## 2 Methods and materials

### 2.1 Patient characteristics

Seven patients diagnosed with histological grade II glioma in our hospital were included between September 2020 and February 2021 in our study. All patients with surgically and oncologically treated glioma previously had a clearly defined indication for glioma treatment under recommendations by the Radiation Therapy and Oncology Group (RTOG) to administer selective radiotherapy. All patients participating in the study had a complete surgical resection. All patients did not have cerebral radiation history. The TOMO plans were designed on a tomotherapy treatment system with 6 MV photon beams and optimized using the least-squares optimization method. VMAT and IMRT plans were designed on the Varian Eclipse treatment planning system with the 6 MV photon beams generated by the Varian IX linear accelerator. The summary of patients, tumor, and treatment details are shown in Table 1. Written evidence of informed consent was obtained from the participant or the participant’s legally acceptable representative.

**Table 1:** Summary of patient, tumor, and treatment details

Patient	Age at diagnosis	Sex	Location	#Resections prior to RT	PTV volume
1	38.4	M	Left temporal lobe	1	198.6 cm^3^
2	39.5	M	Right temporal lobe	1	214.9 cm^3^
3	43.7	F	Left frontal lobe	2	278.5 cm^3^
4	45.2	M	Left temporal lobe	1	310.4 cm^3^
5	39.2	M	Right temporal lobe	1	235.3 cm^3^
6	38.8	F	Right frontal lobe	2	244.9 cm^3^
7	41.9	F	Left temporal lobe	1	204.1 cm^3^


**Informed consent:** Informed consent has been obtained from all individuals included in this study.
**Ethical approval:** The research related to human use has been complied with all the relevant national regulations, institutional policies and in accordance with the tenets of the Helsinki Declaration, and has been approved by the Chongqing University Caner hospital’s ethical review committee (CCH2020-08-14).

### 2.2 CT simulation

A thermoplastic mask and expanded polystyrene were used to fix the patient in the supine position. CT images were obtained by Philips Brilliance Big Bore CT (Philips, Holland) CT with contrast using a 2 mm slice thickness from the vertex of the skull to the mouth floor. Finally, the CT images were transmitted to an Eclipse treatment planning system (Version 8.6, Varian Medical Systems, Palo Alto, CA).

### 2.3 Target volume delineation

The same attending physician contoured each patient’s target areas and normal tissues in the treatment planning system. The gross tumor volume (GTV) include the cyst observed on a gadolinium-enhanced T1 sequence and any non-enhancing abnormality observed on a T2 or fluid-attenuated inversion recovery (FLAIR) sequence in MRI images should be delineated in our LGG cases. Next GTV was added to a 1 cm margin to create the clinical tumor volume (CTV). Finally, CTV was added with a 0.5 cm margin to make the planned tumor volume (PTV). The treatment was performed using a standard fractionation regime in 25 fractions up to a total dose of 50 Gy. The following OARs should be delineated, including lenses, spinal cord, brain stem, optic chiasm, optic nerves, and hypophysis.

RTOG 0615 protocols were adopted for dose constraints in all treatment plans.

### 2.4 Planning process

#### 2.4.1 Dose comparisons and dose-volume histogram

Both RapidArc and IMRT plans were generated with 6 MV photon beam in a linear accelerator equipped with Millennium 120 MLC using the Eclipse treatment planning system. Dose calculation was performed with an anisotropic analytic algorithm. RapidArc plans were devised by the use of two coplanar full arcs. Collimators were rotated from 101 to 201 to minimize the effect of tongue and groove. IMRT plans were devised by using five coplanar beams. TOMO plans were generated with 6 MV photon beam using Tomotherapy Planning Workstation (TomoHD version 1.0.0, Accuray Inc., Sunnyvale, CA). Dose calculation was performed with a superposition or convolution algorithm. The planning parameters were as follows: field width ¼ 2.5 cm, pitch ¼ 0.43, and modulation factor ¼ 3–3.5. To ensure consistency of planning techniques, all treatment plans were devised by physicists with over 3 year clinical experience in IMRT, RapidArc, and Tomotherapy planning. Planning requirements and techniques for planners were also aligned by training, standard protocol, and procedures of the department. Dose-volume histograms (DVHs) were generated and evaluated with the medical physicist until the desired plan was reached. The angles of the rays, the angles of the filters, and the weight ratio were used to optimize the coverage of the planned target volume and to minimize the dose to the OARs. After creating the DVHs and using the required parameters, the radiation Conformity index (CI) was calculated. It is defined as a ratio between the volume covered by the reference isodose, which according to ICRU, is 95% isodose, and the target volume (TV) designated as PTV (equation (1)). A better PTV conformity corresponds to lower values of the CI. The Dose homogeneity index (DHI) is defined as a ratio between the dose reached in 95% of the PTV volume (*D* ≥ 95%) and the dose reached in 5% (*D* ≥ 5%) of the PTV volume (equation (2)). A higher DHI signified poor homogeneous irradiation of the TV.

(1) \text{Conformity}\hspace{.25em}\text{index}{(\text{CI})}_{\text{RTOG}}={V}_{\text{RI}}/\text{TV},]

where *V*_RI_ = Reference isodose volume and PTV = Plan target volume.

(2) \text{Dose}\hspace{.25em}\text{homogeneity}\hspace{.25em}\text{index}(\text{DHI})={D}_{\ge 95 \% (\text{within PTV})}/{D}_{\ge 5 \% (\text{within}\text{PTV})}.]

[14]

**Figure 1:**
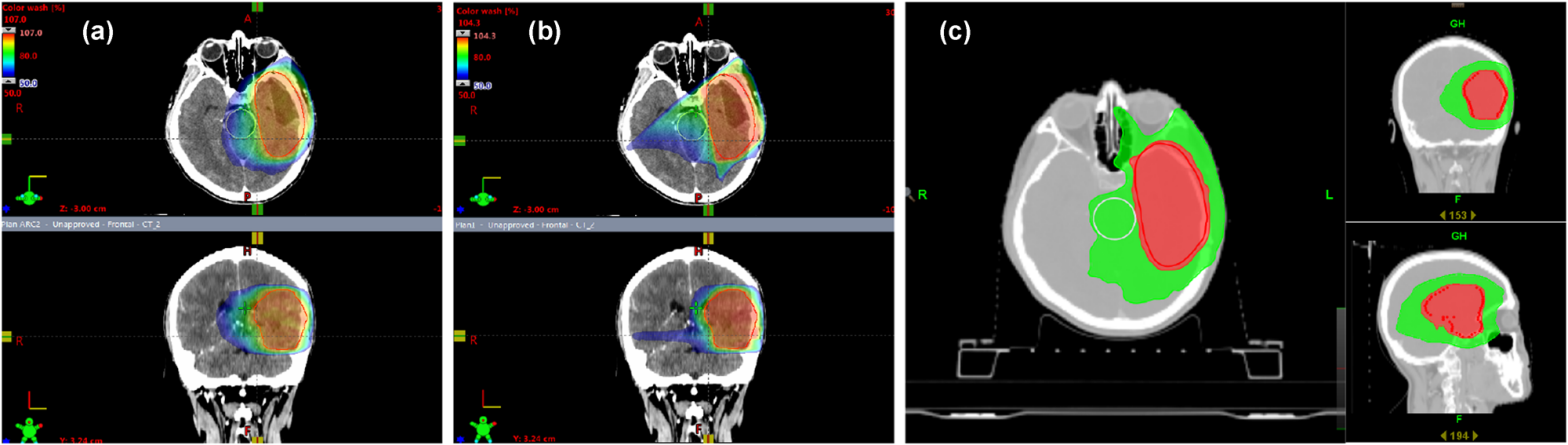
The dose distributions in the grade II gliomas of a typical case, by using VMAT (a), IMRT (b), and tomotherapy (c).

The *D*_98%_ (doses received by 98% of the PTV volume) and *D*_2%_ (doses received by 2% of the PTV volume) were defined as the minimum and maximum doses. The data from the DVHs obtained from all the plans were analyzed. The dose distributions in the grade II gliomas of a typical case, by using VMAT, IMRT, and TOMO are shown in Figure 1.

#### 2.4.2 Organs at risk

Assessment of the *D*_max_ and *D*_mean_ of OARs should be determined according to dose constraints. OARs include the optic chiasm, left eye (L-eye), right eye (R-eye), left lens (L-lens), right lens (R-lens), left optic nerve (L-optic nerve), right optic nerve (R-optic nerve), brain stem, spinal cord, and left and right parotid gland (L-Parotid and R-Parotid).

## 3 Statistical analysis

Nonparametric ANOVA was used to determine if there was a significant difference in any of the parameters examined. *p*＜0.05 was considered statistically significant. SPSS statistical software (SPSS, Chicago, IL) was adopted for analysis.

## 4 Results

### 4.1 PTV coverage

The data analysis of all plans for PTV was performed with the DVH, and the PTV coverage parameters, *D*_98%_, *D*_2%_, *D* mean, CI, and DHI were compared. The study shows that TOMO achieved significantly higher PTV-D_98%_ than VMAT and IMRT (*p* < 0.05) (50.30 ± 0.13 vs 49.21 ± 0.19, *p* = 0.006; 50.30 ± 0.13 vs 49.78 ± 0.18, *p* = 0.014), while there was no difference in terms of PTV-*D*_2%._ The CI was better with IMRT (1.136 ± 0.009) than with TOMO (1.21 ± 0.024) (*p* < 0.05). There was no difference in CI between TOMO (1.21 ± 0.024) and VMAT (1.164 ± 0.014) (*p* > 0.05). TOMO generated a much more favorable DHI than VMAT and IMRT (0.024 ± 0.002 vs 0.041 ± 0.003, *p* = 0.004; 0.024 ± 0.002 vs 0.032 ± 0.003, *p* = 0.027). The mean monitor units (MUs) of TOMO, VMAT, and IMRT were 3,170, 294, and 572. Compared with IMRT, the mean MUs of VMAT were significantly reduced by 48.60 (*p* = 0.000). The data from DVH analysis and MUs are listed in Table 2.

**Table 2:** Plan comparison for PTV and MUs (x ± S)

		TOMO	VMAT	IMRT	*p*		
					TOMO vs VMAT	TOMO vs IMRT	VMAT vs IMRT
PTV	D_98%_ (Gy)	50.30 ± 0.13	49.21 ± 0.19	49.78 ± 0.18	0.006	0.067	0.014
	D_2%_ (Gy)	51.86 ± 0.15	52.09 ± 0.09	51.62 ± 0.19	0.064	0.058	0.079
	D_mean_ (Gy)	51.10 ± 0.15	50.79 ± 0.15	51.60 ± 0.27	0.184	0.186	0.009
	CI	1.21 ± 0.18	1.164 ± 0.014	1.136 ± 0.009	0.052	0.015	0.145
	DHI	0.024 ± 0.002	0.041 ± 0.003	0.032 ± 0.003	0.004	0.027	0.031
	MUs	3,170 ± 97	294 ± 12	572 ± 24	0.000	0.000	0.000

*D*_98%_ = doses received by 98% of the PTV volume, representing the minimum dose of the PTV; D_2%_ = doses received by 2% of the PTV volume, representing the maximum dose of the PTV; CI, conformity index = 95% isodose surface volume divided by the PTV volume; DHI, dose homogeneity index = *D*_≥95% (within PTV)_/*D*_≥5% (within PTV)_.

### 4.2 OARs

In comparison with VMAT and TOMO, IMRT reduced the medium dose of L-lens (*p* = 0.036, *p* = 0.038). Similarly, compared with VMAT, the maximum dose to the R-lens with IMRT was significantly decreased by 21.02% (*p* = 0.026). In addition, compared with TOMO, the maximum dose of the brain stem of IMRT was reduced by 19.29 (*p* = 0.037). The average dose of the OARs is listed in Table 3.

**Table 3:** Dosimetric comparison for OARs (x ± S)

		TOMO	VMAT	IMRT	*p*		
					TOMO vs VMAT	TOMO vs IMRT	VMAT vs IMRT
L-lens	*D*_max_	3.03 ± 0.23	3.28 ± 0.28	3.19 ± 0.37	0.465	0.685	0.677
	*D*_mean_	3.51 ± 0.16	3.54 ± 0.26	2.06 ± 0.15	0.064	0.038	0.036
R-lens	*D*_max_	2.76 ± 0.30	3.33 ± 0.36	2.63 ± 0.34	0.147	0.652	0.021
	*D*_mean_	1.91 ± 0.20	2.55 ± 0.33	1.99 ± 0.24	0.075	0.450	0.081
L-optic nerve	*D*_max_	16.68 ± 4.59	14.51 ± 5.03	13.34 ± 4.66	0.383	0.254	0.026
	*D*_mean_	10.83 ± 2.77	8.09 ± 2.13	6.72 ± 2.05	0.155	0.092	0.153
R-optic nerve	*D*_max_	16.79 ± 5.52	15.56 ± 5.08	16.04 ± 4.89	0.641	0.708	0.652
	*D*_mean_	11.34 ± 3.91	8.63 ± 2.64	7.24 ± 2.00	0.345	0.175	0.131
Optic chiasm	*D*_max_	27.64 ± 4.20	27.80 ± 5.19	25.54 ± 5.01	0.955	0.375	0.112
	*D*_mean_	17.05 ± 4.58	19.44 ± 4.13	18.53 ± 4.45	0.481	0.708	0.545
Hypophysis	*D*_max_	14.85 ± 4.58	19.11 ± 5.64	17.04 ± 5.90	0.275	0.617	0.351
	*D*_mean_	11.92 ± 4.26	15.98 ± 4.59	14.79 ± 5.21	0.281	0.555	0.615
Brain stem	*D*_max_	35.36 ± 4.45	30.48 ± 5.71	28.54 ± 5.11	0.090	0.037	0.105
	*D*_mean_	16.92 ± 5.17	13.41 ± 4.02	13.11 ± 3.90	0.197	0.034	0.065

## 5 Discussion

In the past, there have been comparative dosimetry studies in patients with high-grade glioblastoma. Patients with glioblastoma often have a poor surgical resection, larger postoperative radiation field, higher irradiation dose, and critical organs such as the temporal lobe and brain stem often exceed the maximum tolerated dose. Moreover, patients with high-grade glioblastoma generally have a very short survival time. Long-term radiation-related toxicity is usually not observed at the time of death, so dosimetric comparisons of critical organs are generally of no clinical significance. In conclusion, the dosimetric comparative studies on high-grade glioblastoma are challenging to carry out, have little clinical importance, and are not representative of many LGG patients. Adjuvant postoperative radiotherapy is recommended as the standard treatment for LGG. It can improve the cure rate and prolong the survival time of patients. The importance of radiotherapy for LGG lies in improving the local control rate and protecting the critical organs to allow patients to obtain a higher quality of life. Currently, commonly used radiotherapy methods may have accessibility problems in different countries or regions due to limited economic, equipment, technology, and other reasons. Generally speaking, IMRT is the basis of current precision radiotherapy; VMAT and TOMO need particular technologies and equipment. In addition, the cost for patients with different radiotherapy methods will also be significantly different. The general cost of TOMO will be higher than that of IMRT, especially in China. Therefore, choosing the best benefit radiotherapy for patients with LGG is particularly important. Presently, the therapeutic dose of postoperative radiotherapy has reached a unified consensus, but the choice of the radiation method is still controversial. The pros and cons of IMRT vs other radiotherapy modalities are not clear. Dosimetric comparison of radiation for LGGs in the literature is rare. Our case series study aimed at a comprehensive comparison of dosimetric properties in multiple types of treatment plans and guiding physicians in selecting an appropriate design for the treatment of LGGs.

IMRT radiotherapy’s emergence avoids traditional radiotherapy’s shortcomings enormously in many tumors [15,16]. For example, IMRT improved dose coverage and conformity to the tumor target area for the treatment of glioma and reduced the impact on normal tissues. In addition, in comparison with VMAT, IMRT reduced the medium dose of L-lens [17]. Our study generated similar results that, in contrast with VMAT and TOMO, IMRT reduced the medium dose of L-lens.

Similarly, compared with VMAT, our study indicated that the maximum dose to the R-lens with IMRT was significantly decreased by 21.02%. Compared with TOMO, the maximum dose of the brain stem of IMRT was reduced by 19.29%. IMRT achieved the optimal sparing of nearby critical tissues in the study where the dose of OARs did not exceed the limits among the three treatment technologies.

VMAT, using variable dose rate, gantry rotation speed, and MLC shapes, can deliver good dose coverage and uniformity in the target area. A study on 20 nasopharyngeal carcinomas by Zach et al. demonstrated that VMAT provided better homogeneity, conformity, and sparing of normal tissue than IMRT and a shorter delivery time than TOMO [18]. In another study on ten patients with nasal natural killer/T-cell lymphoma made by Liu et al., compared with IMRT, VMAT had much smaller cold spot volumes and better doses coverage and uniformity to the targets except for fewer MUs and shorter delivery time [19]. While in our study, VMAT was associated with a significant reduction in number of Mus compared with TOMO and IMRT, and there was no significant difference among them in inhomogeneity and conformity. The shortened MUs and consequent delivery time may improve treatment accuracy due to intra‑fractional patient motion. Based on the above data, we believe that VMAT has advantages in applying radiation treatment to head and neck tumors, including glioma.

TOMO is an innovative radiotherapy technique that integrates linear accelerator and CT technology to deliver IMRT in a spiral pattern with a continuously rotating gantry. The integrated image-guidance system provides 3D tumor imaging, achieving precise irradiation, decreasing healthy tissue toxicity, and possible treatment adaption (Image Guided Radiotherapy; IGRT) [20]. In the current study, we found that TOMO achieved significantly higher PTV-*D*_98%_ than VMAT and IMRT, while there was no difference in PTV-*D*_2%_. In addition, TOMO was related to more favorable DHI than VMAT and IMRT.

Compared with VMAT and TOMO, IMRT was first used to treat LGGs. Therefore, IMRT has relatively low requirements for technical personnel and equipment. With the progress of radiotherapy technology, more advanced methods such as VMAT and TOMO have been gradually developed based on IMRT and applied in LGG. Besides the soft hardware and technical difficulties, there is also consideration of economic benefits for radiotherapy methods such as VMAT and TOMO. After all, not every unit can afford TOMO and VMAT equipment and the related software supporting facilities, especially in developing countries like China.

Our study indicated that TOMO, VMAT, and IMRT provide sufficient target dosimetric coverage, and most OARs are sparing clinically. To protect OARs, IMRT is still a better choice for treating patients with grade II gliomas. No single treatment planning method was superior to all others, and a personalized approach is advised for planning and treating LGG patients with radiotherapy. In addition, our study does have some defects. Our single‑institution case series study only included a few LGG patients. The data obtained may not be sufficient enough to draw the above conclusions fully. The included population of our study was only limited to complete resection patients. Patients with R1 or R2 resection may have different dosimetric distributions due to different irradiation doses, so the dosimetric comparison of this population still needs further detailed and profound research to clarify.
